# Health promotion in primary and secondary schools in Denmark: time trends and associations with schools’ and students’ characteristics

**DOI:** 10.1186/s12889-015-1440-z

**Published:** 2015-02-07

**Authors:** Kirsten Nabe-Nielsen, Rikke Krølner, Laust Hvas Mortensen, Marie Birk Jørgensen, Finn Diderichsen

**Affiliations:** Department of Public Health, Section of Social Medicine, University of Copenhagen, Øster Farimagsgade 5, Copenhagen, 1014 Denmark; The National Institute of Public Health, University of Southern Denmark, Copenhagen, Denmark; The National Research Centre for the Working Environment, Copenhagen, Denmark

**Keywords:** Alcohol, Anti-smoking, Bullying, Diet, Physical activity, Sex education

## Abstract

**Background:**

Schools are important arenas for interventions among children as health promoting initiatives in childhood is expected to have substantial influence on health and well-being in adulthood. In countries with compulsory school attention, all children could potentially benefit from health promotion at the school level regardless of socioeconomic status or other background factors. The first aim was to elucidate time trends in the number and types of school health promoting activities by describing the number and type of health promoting activities in primary and secondary schools in Denmark. The second aim was to investigate which characteristics of schools and students that are associated with participation in many (≥3) versus few (0–2) health promoting activities during the preceding 2–3 years.

**Methods:**

We used cross-sectional data from the 2006- and 2010-survey of the *Health Behaviour in School*-*aged Children* study. The headmasters answered questions about the school’s participation in health promoting activities and about school size, proportion of ethnic minorities, school facilities available for health promoting activities, competing problems and resources at the school and in the neighborhood. Students provided information about their health-related behavior and exposure to bullying which was aggregated to the school level. A total of 74 schools were available for analyses in 2006 and 69 in 2010. We used chi-square test, *t*-test, and binary logistic regression to analyze time trends and differences between schools engaging in many versus few health promoting activities.

**Results:**

The percentage of schools participating in ≥3 health promoting activities was 63% in 2006 and 61% in 2010. Also the mean number of health promoting activities was similar (3.14 vs. 3.07). The activities most frequently targeted physical activity (73% and 85%) and bullying (78% and 67%). Schools’ participation in anti-smoking activities was significantly higher in 2006 compared with 2010 (46% vs. 29%). None of the investigated variables were associated with schools’ participation in health promoting activities.

**Conclusion:**

In a Danish context, schools’ participation in health promotion was rather stable from 2006 to 2010 and unrelated to the measured characteristics of the schools and their students.

## Background

Schools are important arenas for interventions among children as health promoting initiatives in childhood is expected to have substantial influence on health and well-being in adulthood [1-3]. In countries with compulsory school attention, all children could potentially benefit from health promotion at the school level regardless of socioeconomic status or other background factors [4,5].

In a Swedish 1990-survey, it was reported that the vast majority of schools (94–99%) taught about alcohol, sex, smoking, drugs, physical health/exercise, and bullying/violence to all students during their school career [6]. Nevertheless, despite the obvious advantages of health promotion at the school level, the literature on the frequency of different types of health promotion in primary and secondary schools is sparse. Most studies describe proportions of schools with activities and policies directed towards a specific behavior [7-10], and only one of these previous studies [9] combined variables measured at the student level and the school level.

Furthermore, only few studies have described what characterizes schools that engage in health promotion. Factors that have been investigated are school compositional characteristics (e.g. number of students or percentage of White students), and socioeconomic indicators (e.g. discretionary dollars per pupil) [11,12]. None of these studies were conducted in Scandinavia, and therefore we wanted to investigate whether schools’ engagement in health promoting activities also related to these school characteristics (i.e., school size, ethnic composition, and affluence) in a Danish context.

Also the social environment/school climate and headmasters’ attitudes towards the school food environment has been mentioned as crucial factors for health promotion at the school level, along with a health enhancing physical environment, e.g. access to healthy food choices in the school canteen or necessary facilities for sports [8,13,14]. Therefore, we also wanted to investigate how measures of the social climate among teachers and students, respectively, and the availability of school facilities for healthy food choices and physical activity, were related to schools’ engagement in health promoting activities.

We also argue that competing problems at the school and in the local area, e.g. in terms of sick leave, truancy, crime, and vandalism would be given priority over health promoting activities. For this reason, we expect that the occurrence of such competing problems would reduce the frequency of health promoting activities although such problems are likely to appear hand in hand with poorer health-related behaviors. In contrast, we expect that a socially and economically resourceful neighborhood would be associated with a higher frequency of health promoting activities in addition to the school curriculum.

Finally, students’ characteristics may both be indicative of the need for health promoting activities (i.e. unhealthy behaviors) and a result of such activities (i.e. healthy behaviors). We employ a rather broad health concept, as we include both’classic’ risk factors (diet, smoking, alcohol, and physical activity), a risk factor for sexually transmitted diseases (sexual intercourse), and a risk factor for poor mental health (exposure to bullying).

Knowledge of the prevalence of schools’ health promoting activities and characteristics of schools initiating health promoting activities is of relevance to public health practice and research. First, knowledge about the prevalence and types of health promotion in different time periods will elucidate time trends in health promotion at the public school level. Second, knowledge about what characterizes schools that are less engaged in health promotion increases the possibility of targeting such schools directly to ensure that relevant health promoting activities are initiated. Third, knowledge on differences between schools engaging in many and few activities shed empirical light on the potential selection of schools into intervention and reference groups when studying the effect of health promoting interventions in observational and quasi-experimental designs.

The first aim of the study was to elucidate time trends in the number and types of school health promotion activities. The second aim of the study was to analyze whether the engagement in many (≥3) versus few (0–2) health promoting activities during the preceding 2–3 years was associated with school compositional characters, school facilities available for health promoting activities, competing problems and resources at the school and in the neighborhood, and the students’ characteristics in terms of dietary habits, level of physical activity, smoking, binge-drinking, sexual habits, and exposure to bullying.

## Methods

### Study design and study population

We used Danish data from the international, WHO-coordinated *Health Behaviour in School*-*aged Children* (HBSC) study [15,16]. We received permission to use data from the steering committee of the Danish part of the HBSC study. HBSC is a cross-sectional study conducted every fourth year among 11-, 13-, and 15-year old children. The Danish study population consisted of 5th, 7th, and 9th-graders in a random sample of Danish schools. The data consisted of self-completion questionnaires for the students administered in the classroom and a questionnaire filled in by the headmaster of each school.

We used data from 2006 (participating schools: n = 80; school participation rate 80%; participating students: n = 6269; response rate: 89%) and from 2010 (participating schools: n = 75; school participation rate: 55%; participating students, n = 4,922; response rate: 86%). Only schools with valid information on the outcome (2006: n = 74 and 2010: n = 69) were included in the analyses.

### Ethics

Invitations to participate were sent to the school boards, schools’ headmaster, and school councils. The school boards consist of parents’ representatives and they approved the students’ participation on behalf of the parents; no informed consent for each individual student was obtained. If the school volunteered, the students filled in the questionnaire during school hours. The students were informed that participation was completely voluntary and anonymous and no identity information (e.g. name or birthday) should be provided. Questionnaires were returned in a closed envelope and only the research group had access to the questionnaires.

### Participation in health promoting activities

In 2006 *participation in health promoting activities* was based on the headmasters’ responses to the question: ‘Within the past three years, has your school participated in projects with the following content…?’ and in 2010 the headmasters were asked: ‘During the last couple of years, has the school made a special effort in relation to or participated in projects with the following content…?’. The response options in 2006 and 2010 were ‘Healthy school network’, ‘Physical activity’, ‘Diet’, ‘Prevention of bullying and/or violence’, ‘Anti-smoking’, and ‘Sex education’. In 2006, the response option ‘Alcohol and/or drug abuse’ was also included.

We calculated the total number of activities that each school engaged in (range 0–6; to obtain comparability, the response option ‘Alcohol and/or drug abuse’ was excluded in the sum as this information was only available for 2006). We dichotomized the sum variable by the median into *few* health promoting activities (0–2) and *many* health promoting activities (≥3). As we did not have a predefined definition of what constitutes *few* and *many* health promoting activities, the median was chosen as cut-point in order to obtain two groups of approximately equal size.

### Schools’ and students’ characteristics

#### Questionnaire for headmasters

*School compositional characteristics*: Number of students, number of teachers, and proportion of ethnic minorities. Headmasters’ responses to the first two variables were verified against official statistics.

*Facilities for health promotion*: ‘Below, you’ll find a list of facilities. Please, indicate to what extent the facilities at your school match the needs of the school’. The facilities included in the present study were ‘Outdoor areas’, ‘Access to foods (2006)’/‘Access to healthy food’ (2010), ‘Dining hall’, ‘Gym/sports center, and ‘Sport equipment’. The response options were dichotomized into ‘Correspond to a large extent to the needs of the school’ and ‘Correspond to some extent to the needs of the school/Do not correspond to the needs of the school’.

*Competing problems at the school* (*2010*): ‘Think about the school. Do you agree or disagree with the following statements?’ 1) There are problems with students playing truant; 2) ‘There are problems with conflicts among the students’; 3) ‘There are problems with conflicts among the teachers’; 4) ‘There are problems with sick leave among the teachers’; 5) ‘There are many teachers on long-term sick leave’; 6) ‘There are problems with vandalism or graffiti in the school neighborhood’; 7) ‘This is a popular school that many families want their children to attend’. The four response categories ranged from ‘Totally disagree’ (=1) to ‘Totally agree’ (=4) (the 7th item was coded in reverse), and a sum score was calculated.

*Competing problems in the school*’*s neighborhood* (*2010*): ‘Think about the neighborhood, where the school is situated. Do you agree or disagree with the following statements?’ 1) ‘There are trash and shards of glass that lie about‘; 2) There are problems with crime in the area’; 3) There are problems with distribution of hashish and other drugs’. The four response categories ranged from ‘Totally disagree’ (=1) to ‘Totally agree’ (=4), and a sum score was calculated.

*Social climate (2010)*: ‘How do you rate the social climate among teachers?’ and ‘How do you rate the social climate among students?’. The response categories were ‘Very good’ , ‘Good’ , ‘Fairly good’ , ‘Poor’ , and ‘Very poor’.

*Resources in the school neighborhood*: ‘Think about the area, where the school is situated. Do you agree or disagree with the following statements?’ 1) ‘Adults in the neighborhood intervene if groups of young people make trouble outside the school property’; 2) ‘Many affluent people live here’; 3) ‘It is an attractive area, where many people want to live’; 7) ‘The area is safe for traffic’. The four response categories ranged from ‘Totally disagree’ (=1) to ‘Totally agree’ (=4), and a sum score was calculated.

*Affluence*: ‘How affluent is the neighborhood, where the school is situated?’ followed by five response categories trichotomized into ‘Very affluent’/‘Somewhat affluent’, ‘Like the average’, and ‘Not at all affluent’/‘Not so affluent’.

#### Questionnaire for students

*Intake of fruit*, *vegetables and unhealthy snacks*: A food frequency questionnaire was used, and the students were asked: ‘How many times a week do you usually eat or drink…?’, with seven response categories ranging from ‘Several times every day’ to ‘Never’. When deciding on cut points we considered both the dietary recommendations from the Danish Veterinary and Food Administration under the Ministry of Food, Agriculture and Fisheries [17] and the actual distribution of the variables. For intake of fruit and vegetables, responses categories were dichotomized into ‘Less than daily’ versus ‘Daily’. For ‘Sweets’ and ‘Coke or other soft drinks that contain sugar’ we dichotomized into ‘Less than weekly’ versus ‘Weekly’.

*Leisure*-*time physical activity*: ‘Outside school hours: How often do you usually exercise in your free time so much that you get out of breath or sweat?’ The seven response categories were ‘every day’, ‘4–6 times a week’, ‘2–3 times a week’, ‘Once a week’, ‘Once a month’, ‘Less than once a month’, and ‘Never’. The variable was dichotomized into ‘4 times a week or more’ versus ‘Less than 4 times a week’. The choice of cut-point (i.e., ≥4 times/week vs. <4 times/week) was determined in order to obtain two groups of similar size while at the same time—as far as possible—adhering to the guidelines for vigorous physical activity among children. Smoking habits: ‘How often do you smoke tobacco at present?’ and the four response categories were dichotomized into ‘Non-smoker’ and ‘Occasional/daily smoker’.

*Bullying*: ‘How often have you been bullied at school in the past couple of months?’ The five response categories were dichotomized into ‘Never’ and ‘At least once or twice’.

*Binge drinking (only 9th-graders)*: ‘Think about the past 30 days: How many times have you been drinking five or more drinks in rapid succession?’ The response options were dichotomized into ‘None’ and ‘At least once’.

*Sexual intercourse* (*only 9th*-*graders*): ‘Have you ever had sexual intercourse?’ The response options were ‘No’ and ‘Yes’.

#### Statistical analyses

All variables measured at the individual (i.e. student) level were aggregated to the school level to obtain variables that expressed the proportion (percentage) at each school that ate fruit daily, ate vegetables daily, etc.

**Figure 1:**
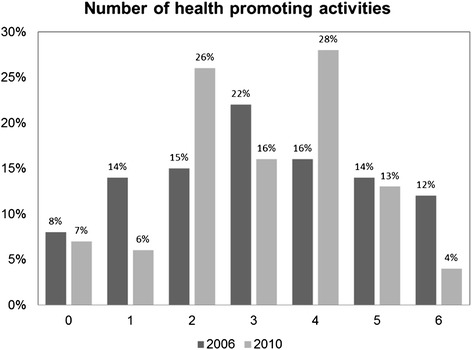
**Percentage of Danish HBSC schools participating in one to six health promoting activities in 2006 and 2010.**

**Figure 2:**
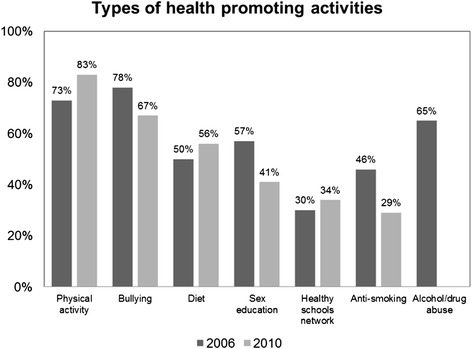
**Percentage of Danish HBSC schools participating in specific types of health promoting activities in 2006 and 2010.** The item on schools’ participation in activities targeting alcohol/drug abuse was not included in the 2010 questionnaire.

First, we graphically presented the distribution of the total number and the type of health promoting activities (Figures 1 and 2).

Second, we analyzed differences in the distribution of the independent variables between 2006 and 2010 (χ^2^ test for categorical variables; *t*-test for continuous variables) (Table 1).

**Table 1:** **The table shows the differences between Danish HBSC schools in 2006 and 2010**

		**Year**		
		**2006**	**2010**	**p** **-** **value**
		**(n**=**74)**	**(n**=**69)**	
**School compositional characteristics**				
	Number of students at the schools (mean, SD)	374 (202)	304 (215)	0.157
	Number of teachers at the schools (mean, SD)	33 (19)	28 (19)	0.124
	Number of students per teacher (mean, SD)	11 (2)	11 (2)	0.890
	Ethnic minorities (mean %, SD)	11 (20)	7 (14)	0.251
	Number of grades included in the study (%)			
	5^th^ grade	66 (89%)	54 (78%)	0.075
	7^th^ grade	61 (82%)	60 (87%)	0.454
	9^th^ grade	21 (28%)	24 (35%)	0.410
**Facilities for health promotion projects**				
	Number of schools indication that the facilities correspond to the school’s needs (n,%)			
	Outdoor areas	35 (47%)	35 (51%)	0.682
	Access to (healthy) foods	14 (19%)	15 (23%)	0.579
	Dining hall	9 (12%)	6 (9%)	0.519
	Gym or sports center	50 (68%)	29 (43%)	**0.003**
	Sports equipment	37 (50%)	21 (31%)	**0.021**
Behavioral characteristics of the students*				
	Fruit daily (mean %, SD)	43% (9)	50% (11)	**<** **0.001**
	Vegetables daily (mean %, SD)	37% (9)	42% (12)	**0.003**
	Sweets ≤1 time/week (mean %, SD)	42% (11)	49% (13)	**0.003**
	Soft drinks ≤1 time/week (mean %, SD)	57% (11)	60% (12)	**0.047**
	Physically active ≥4 times/week (mean %, SD)	41% (9)	34% (9)	**<** **0.001**
	Non-smokers (mean %, SD)	91% (6)	92% (6)	**0.040**
	Not been bullied (mean %, SD)	74% (9)	79% (8)	**0.001**
	No binge-drinking (mean %, SD)**	44% (18)	69% (14)	**<** **0.001**
	No sexual intercourse (mean %, SD)**	62% (13)	62% (13)	0.756

*Expressed as mean percentage, calculated as the sum of the percentage of students eating fruit daily at each school divided by the number of schools, and similarly for the remaining behavioral characteristics.

**Only obtained from 9th graders.

Significant values are written in bold face.

Third, we analyzed the distribution of school and student characteristics among schools participating in many (≥3) versus few (0–2) health promoting activities (χ^2^ test for categorical variables; *t*-test for continuous variables). The analyses were conducted separately for 2006- and 2010-data (Table 2).

**Table 2:** **Differences between schools that participate in few** (**0**–**2**) **and many** (≥**3**) **health promoting activities during the preceding 2**–**3 years**

	**2006** **(n** **=** **74)**			**2010** **(n=** **69)**				
	**Number of health promoting activities**							
	**0**-**2** **(n** **=** **27)**	≥**3** **(n** **=** **47)**	**p-** **value**	**0-** **2** **(n=** **27)**	≥**3** **(n=** **42)**	**p-** **value**	**OR** ^**†**^	**95%** **CI** ^**††**^
**School compositional characteristics**								
Number of pupils at the schools (mean, SD)	381 (208)	339 (200)	0.395	264 (184)	329 (232)	0.232	1.0	0.99-1.0
Number of teachers at the schools (mean, SD)	36 (20)	31 (19)	0.280	25 (17)	29 (23)	0.350	1.0	0.98-1.0
Number of students per teacher (mean, SD)	11 (2)	11 (3)	0.331	11 (2)	11 (3)	0.847	1.1	0.9-1.2
Ethnic minorities (mean %, SD)	11% (21)	10% (20)	0.931	4% (6)	9% (16)	0.064	1.0	0.99-1.0
**Facilities for health promotion projects**								
School facilities correspond to the school’s needs								
Outdoor areas	48%	47%	0.912	44%	55%	0.403	0.8	0.4-1.7
Access to (healthy) food	22%	17%	0.582	15%	28%	0.202	0.8	0.3-1.9
Dining hall	19%	9%	0.218	7%	10%	0.715	1.5	0.6-4.3
Gym or sports center	78%	62%	0.155	33%	49%	0.208	1.0	0.5-2.0
Equipment for sports	52%	49%	0.809	30%	32%	0.856	1.0	0.5-2.0
**Competing problems**								
Problems at the school (mean score, SD)				12 (3)	11 (3)	0.568	0.9	0.8-1.1
Problems in the neighborhood (mean score, SD)				10 (2)	9 (2)	0.604	0.9	0.7-1.2
**Resources**								
Social climate among teachers						0.833		
Very good				56%	52%		1.0	
Good				41%	41%		1.1	0.4-2.9
Fairly good				4%	7%		2.0	0.2-21.6
Social climate among students^€^						0.838		
Very good				22%	29%		1.0	
Good				67%	62%		0.7	0.2-2.3
Fairly good				11%	10%		0.7	0.1-4.0
Resources in the local area (mean score, SD)				11 (2)	12 (2)	0.453	1.1	0.8-1.5
Affluence of school neighborhood						0.471		
Very/somewhat affluent				19%	13%		1.0	
Like the average				56%	48%		1.3	0.3-5.2
Not at all/not so affluent				26%	40%		2.3	0.5-10.5
Behavioral characteristics of the students*								
Fruit daily (mean %, SD)	42% (10)	43% (8)	0.723	51% (12)	49% (11)	0.741	1.0	0.97-1.03
Vegetables daily (mean %, SD)	36% (10)	38% (9)	0.370	43% (11)	43% (12)	0.710	1.0	0.98-1.05
Sweets ≤1 time/week (mean %, SD)	39% (12)	44% (10)	0.047	48% (11)	48% (15)	0.877	1.0	0.99-1.04
Soft drinks ≤1 time/week (mean %, SD)	55% (14)	57% (9)	0.510	58% (12)	62% (13)	0.162	1.0	0.99-1.1
Physically active ≥4 times/week (mean %, SD)	39% (8)	42% (10)	0.168	35% (9)	33% (9)	0.385	1.0	0.98-1.04
Non-smokers (mean %, SD)	90% (7)	91% (5)	0.485	94% (6)	92% (7)	0.403	1.0	0.9-1.1
Not been bullied (mean %, SD)	76% (8)	74% (9)	0.374	80% (8)	78% (8)	0.424	1.0	0.9-1.02
No binge-drinking (mean %, SD)**	45% (15)	43% (20)	0.707	71% (20)	68% (15)	0.530	1.0	0.98-1.01
No sexual intercourse (mean %, SD)**	65% (12)	60% (14)	0.182	59% (12)	64% (14)	0.232	1.0	0.97-1.03

^€^None of the participants used the response options ‘Poor’ or ‘Very poor’.

*Expressed as mean percentage, calculated as the sum of the percentage of students eating fruit daily at each school divided by the number of schools, and similarly for the remaining behavioral characteristics.

**Only obtained from 9th graders.

^†^OR = Odds ratio. The ORs express the association between each of the independent variables and the participation in many (≥3) health promoting activities.

^††^95% CI = 95% Confidence Intervals.

Fourth, we used binary logistic regression to provide odds ratios (OR) and 95% confidence intervals (95% CI) for the association between participation in health promoting activities and the schools’ and students’ characteristics (Table 2). In these analyses, data for 2006 and 2010 were collapsed, except for the measures of ‘competing problems’ and ‘resources’ as these were only available in 2010.

## Results

School characteristics of the 2006- and 2010-samples did not differ significantly. Still, in the 2010-sample we found a lower frequency of having access to a gym/sports center (68% vs. 43%, p = 0.003) and equipment for sports (50% vs. 31%, p = 0.021) (Table 1).

Whereas the dietary habits of the pupils were better in the 2010-sample the level of physical activity was worse. The mean proportion of non-smokers and students not reporting bullying was a bit higher in 2010 compared with 2006. The mean proportion of 9th-graders who had not been binge-drinking was remarkably higher in 2010 compared with 2006 (Table 1).

Figure 1 shows that in 2010, fewer schools engaged in 0–1 (22% vs. 13%) or 5–6 (26% vs. 17%) health promotion activities while more schools participated in either 2 or 4 health promoting activities compared with 2006. The mean number of activities per school did not differ between 2006 and 2010 (3.14 vs. 3.07; p = 0.824).

Figure 2 shows that the types of health promoting activities did not differ substantially between 2006 and 2010 with the exception of anti-smoking activities which was 46% in 2006 and 29% in 2010 (p = 0.044). Most schools (73–83%) reported that they had participated in activities to increase the physical activity level among students, and 67–78% reported that they had engaged in anti-bullying activities. About half of the schools (41–57%) participated in activities focusing on diet and sex education, and one third of the schools were members of the healthy schools network. In 2006, 65% of the schools reported that they had initiated activities to reduce alcohol/drug abuse.

We found no statistically significant differences between schools that were engaging in 0–2 versus ≥3 health promoting activities. The only exception was a slightly higher proportion of students that seldom ate sweets (<1 times/week) in schools participating in many health promoting activities in 2006 (Table 2).

To perform a *post hoc* investigation of the sensitivity of the results in relation to the choice of statistical method, we performed the same analyses using a general linear model with number of health promoting activities as continuous outcome. The results of the general linear model mimicked the results of the binary logistic regression. Thus, the results are rather robust in relation to the statistical method used.

## Discussion

### Primary findings

The mean number of the schools’ health promoting activities was similar in 2006 and 2010 (approximately 3 activities during the preceding 2–3 years). The most frequent activities targeted physical activity and bullying, and the types of health promotion were similar, except that anti-smoking activities were more frequent in 2006 compared with 2010. The changes in activities targeting alcohol and/or drug abuse could not be estimated as this information was only available in 2006. None of the investigated characteristics of schools and students were associated with the schools’ engagement in health promoting activities.

### Comparison with previous studies

In the present study, 50–56% of the sample participated in diet-related activities, and 73–83% of the sample participated in physical activity-related activities. Another Danish study (1999-data) found that 3% of Danish schools had a written school policy on nutrition [7]. In a study from Minnesota (2001-data), 32% of the participating schools had a school policy about nutrition and food [8]. A study from Belgium-Flanders (2003-data) reported that whereas most primary schools (89–97%) had informal or written rules to restrict the consumption of biscuits, sweets, and savory snacks this was only the case among half of the secondary schools [9]. Finally, a study from the Netherlands (2006-data) reported that 15% of the schools had a policy on healthy nutrition, and 3% had a policy on overweight prevention. In contrast, a majority of the schools (60–85%) reported having taken initiative to improve healthy eating behavior and increase the level of physical activity among children [10]. We have no comparisons for the remaining types of health promotion. Still, the findings from the present study are not directly comparable with results from previous research mainly due to differences in outcome measures.

Our finding of no association between the schools’ and students’ characteristics and the schools’ participation in health promotion activities contrasts findings from the US were the number of health policies and programs was positively related to the schools’ discretionary dollars per pupil, the size of enrollment and with the percentage of White students [12]. More in line with our study, another study reported that the proportion of ethnic minorities was not associated with the participation in health promoting activities [11]. Still, the comparability of U.S. schools and Danish schools is questionable. Studies from the U.S. include activities such as the designation of a weapons- and drug-free school zone and prohibiting tobacco advertisement on school building and grounds as part of an evaluation of the promotion of a healthy, physical school environment [11]. This appears to be very far from the Danish context, and also determinants of health promotion may operate differently in different countries.

We did not find a significant association between the aggregated measures of the students’ characteristics and the frequency of health promoting activities at the school level. There are several potential explanations for this finding. First, as mentioned in the introduction students’ characteristics may be both a cause (i.e. unhealthy behaviors) and a consequence (i.e. healthy behaviors) of schools’ engagement in health promoting activities and thus any differences may level out. Second, it may be speculated that schools’ health promoting activities are initiated because of political incentives and to promote a good reputation. Third, low statistical power may be contributing to the lack of statistically significant differences between the groups. However, this is not likely to be an important explanation as the figures show that the students’ characteristics are rather similar when comparing schools engaging in few and many health promoting activities. Fourth, although we aimed at analyzing students’ characteristics directly related to the outcomes under study, i.e. dietary habits, level of physical activity, smoking, binge-drinking, sexual habits, and exposure to bullying, we may not have captured the characteristics of the students relevant for school’s engagement in health promoting activities.

Nevertheless, the crucial factor is, whether the initiatives reach the students and improve their health and health behaviors, which is possible under some circumstances [18-22]. In the present study, we found no association between students’ characteristics and the frequency of schools’ health promoting activities. The interpretation may either be that students’ characteristics do not affect schools’ participation in health promoting activities or that participation in health promoting activities has improved the characteristics of the students so they reached the same level as the students at other schools.

### Strengths and limitations

The strengths of the study lie within its combination of data obtained from students and headmasters. Furthermore, invited schools were a randomly drawn sample of Danish schools, and the repeated surveys including questions (with modifications, though) about participation in health promoting activities made it possible for us to investigate time trends in schools’ engagement in such activities.

A limitation of the study is its small effective sample size (74 schools in 2006 and 69 schools in 2010) implying a limited statistical power. Disregarding statistical significance there appeared to be no convincing systematic differences between the schools that participated in many versus few health promoting activities.

Another important limitation is the cross-sectional design, and we cannot elucidate the complicated mechanisms that are likely to determine whether schools participate in health promoting activities. The preclusion of causal interferences is especially relevant regarding the relation between health promoting activities and the students’ characteristics as these obviously can be both a cause and a consequence of the schools’ health promoting activities whereas this bidirectional relationship is less likely with respect to, for example, the affluence of the neighborhood.

The school response rate was lower in 2010 compared with 2006 (55% vs. 80%) which gives rise to concerns regarding the comparability of the results of the two surveys. In both survey years, lack of time and participation in other activities or studies were often indicated as the reason for non-participation [23,24]. Therefore, schools participating in many activities (health-related or not) were less inclined to participate in HBSC, which would result in an underestimation of the frequency of schools’ participation in health promotion, especially in 2010. However, in general the participation in specific types of health promotion was similar in the two surveys. Furthermore, we did not find a statistically significant difference in the mean number of activities that the schools were participating in, which rather indicate a lower response rate in both extremes (i.e. those that participate in 0–1 and 5–6 activities).

The slight changes in the questions in the headmasters’ questionnaire may have influenced the comparability of the data over time (e.g. with respect to the facilities for health promotion the response option ‘access to food’ (2006) changed to ‘access to healthy food’ (2010); the time frame for health promoting activities; and omission of the response option ‘alcohol/drug abuse’). Also, headmasters’ responses to the questions about their school’s participation in health promotion may be subject to a wide variety of individual interpretation: Health promoting activities could imply anything from a short information campaign to comprehensive multi-faceted projects, and the health enhancing effects of a few comprehensive projects may be greater than the effect of (many) small projects. However, the headmasters were not asked to provide further details about the activities. The validity of the measure would have been improved, for example, by explicitly defining what is meant by the term ‘project’ and by being more precise about the time frame (especially the term ‘last couple of years’, which was used in 2010, is open for individual interpretation). However, to grasp the potential comprehensiveness of schools’ participation in health promotion, questions about specific activities, duration of the projects, participants, and methods need to be inquired.

## Conclusion

The present study showed that the frequency of health promoting activities in Danish schools have been rather stable during the period 2006 to 2010, except for a lower participation in anti-smoking project in 2010 compared with 2006. Bullying and physical activity are the areas that receive most attention. The participation in activities targeting physical activity was at about the same level in 2006 and 2010, although the necessary facilities for physical activity appeared to be less available in 2010 compared with 2006.

In a Danish context, school compositional characteristics, availability of facilities for health promotion activities, competing problems and resources at the school and in the neighborhood, and the measured characteristics of the students were not associated with schools’ participation in health promoting activities. Thus, this study does not support the hypothesis that schools’ and students’ characteristics are associated with the schools’ participation in health promoting activities.

### Perspectives

With reference to the study’s relevance to public health practice and research as mentioned in the introduction, we find no signs of an alarming decrease in engagement in health promotion at the school level which warrants immediate action. However, the (significant) decline in anti-smoking activities and the (insignificant) decline in sex education indicate that future attention is necessary. As we did not find convincing predictors of participation in health promoting activities the results of the present study cannot be used for selecting schools that are in need of e.g. instrumental or economical support in order to initiate health promotion. Cross-cultural studies with comparable data from different countries may determine whether the similarities between the schools, as observed in the present study, are due to the societal context within which the Danish schools operate. Finally, as this study does not support a hypothesis of a strong selection into health promoting activities, we suggest that this concern should not keep researchers from initiating non-randomized intervention studies in the future while still taking appropriate account of potential confounding factors.
